# Arthroscopic vs. open Broström-Gould for repairing anterior talofibular ligament: mid-term outcomes comparison

**DOI:** 10.3389/fsurg.2023.1181493

**Published:** 2023-05-10

**Authors:** Ji Wang, Zhihong Tang, Hao Sun, Jing Lv, Hongyang Jiang, Yong Yue

**Affiliations:** Department of Orthopaedics, No. 967 Hospital of the PLA Joint Logistics Support Force, Dalian, China

**Keywords:** anterior talofibular ligament, ankle instability, arthroscopy, open Broström-Gould procedure, treatment

## Abstract

**Introduction:**

There have been few mid-term follow-up studies comparing arthroscopic and open Broström-Gould repair of the anterior talofibular ligament (ATFL). The purpose of this study was to evaluate the mid-term therapeutic effectiveness of arthroscopic ATFL repair with open Broström-Gould repair for chronic lateral ankle instability.

**Methods:**

We retrospectively reviewed the database of patients with chronic lateral ankle instability undergoing repair of the ATFL from June 2014 to June 2018. The choice of surgical approach will depend on computer-generated randomization. In total, 49 patients underwent the arthroscopic Brostrom-Gould technique (group AB), while the other 50 patients underwent the open Broström-Gould technique (group OB). The surgery duration, hospitalization time, postoperative complications, the preoperative/postoperative manual anterior drawer test (ADT), Visual analog scale (VAS) scores, American Orthopaedic Foot & Ankle Society (AOFAS) scores, Karlsson-Peterson (K-P) scores, and Tegner activity scores were collected for comparative analysis during the follow-up period of 48 months.

**Results:**

At the final follow-up, the clinical outcomes, including ADT, VAS, AOFAS, K-P, and Tegner activity scores, were significantly improved after either arthroscopic or open treatment. Specifically, the AOFAS and K-P scores in the group AB were significantly higher than those in the group OB at 6 months post-surgery (*P* < 0.05). Additionally, there were no significant differences in other clinical outcomes and postoperative complications between the two groups.

**Conclusions:**

Arthroscopic has predictable and good mid-term results after ATFL and may be a secure and effective alternative to open Broström-Gould repair.

## 1. Introduction

The anterior talofibular ligament (ATFL) is the one of most frequently injured ligaments in the ankle sprains with an increasing incidence (1, 2). Most of these ankle sprains can be treated effectively with conservative methods. However, about 10%–20% develop chronic lateral ankle instability and consequently require surgical ATFL repair (3). As is well-known, the open Broström technique and its modifications are regarded as the gold standard surgical approach for repairing ATFL (4, 5). Various surgical procedures for repairing ATFL have been reported in recent years, ranging from open repair to limited open repair or percutaneous repair, and arthroscopic repair (5–7). The anterior talofibular ligament is increasingly being repaired arthroscopically (2). However, studies directly comparing outcomes of arthroscopic and open Broström-Gould ATFL repair remain to be controversial. Multiple studies suggest that open Broström-Gould repair and arthroscopic repair of the ATFL have comparable therapeutic efficacy for chronic lateral ankle instability (4, 8, 9). Ulrike Wittig et al. performed systematic review using one RCT (Randomized controlled trial) and seven RCSs (Retrospective comparative studies) including a total of 420 patients with AFTL injury to prove that clinical outcomes did not differ substantially between patients treated with either arthroscopic or open Broström repair (10). The study of Bo Jun Woo et al. indicated the arthroscopic Brostrum-Gould technique produced better clinical outcomes than the open technique at 12 months of follow-up (11). However, previous studies have shown that arthroscopy has higher complication rate, which mainly include sensitive nerve damage (12, 13). The conclusions of the published articles are still inconsistent, and most of the studies mainly focus on the short-term effect of objective direct repair to ATFL for chronic lateral ankle instability, with a small sample. Therefore, this is necessary to compare outcomes for the two techniques with a relatively long follow-up period. The objective of this article was to investigate the outcomes of arthroscopic vs. open Broström-Gould, including surgery duration, hospitalization time, postoperative complications, the preoperative/postoperative ADT, VAS scores, AOFAS scores, K-P scores, and Tegner activity scores, to prove whether arthroscopic surgery is superior to open surgery in the mid-term follow-up period.

## 2. Method

### 2.1. Patients

**Figure 1:**
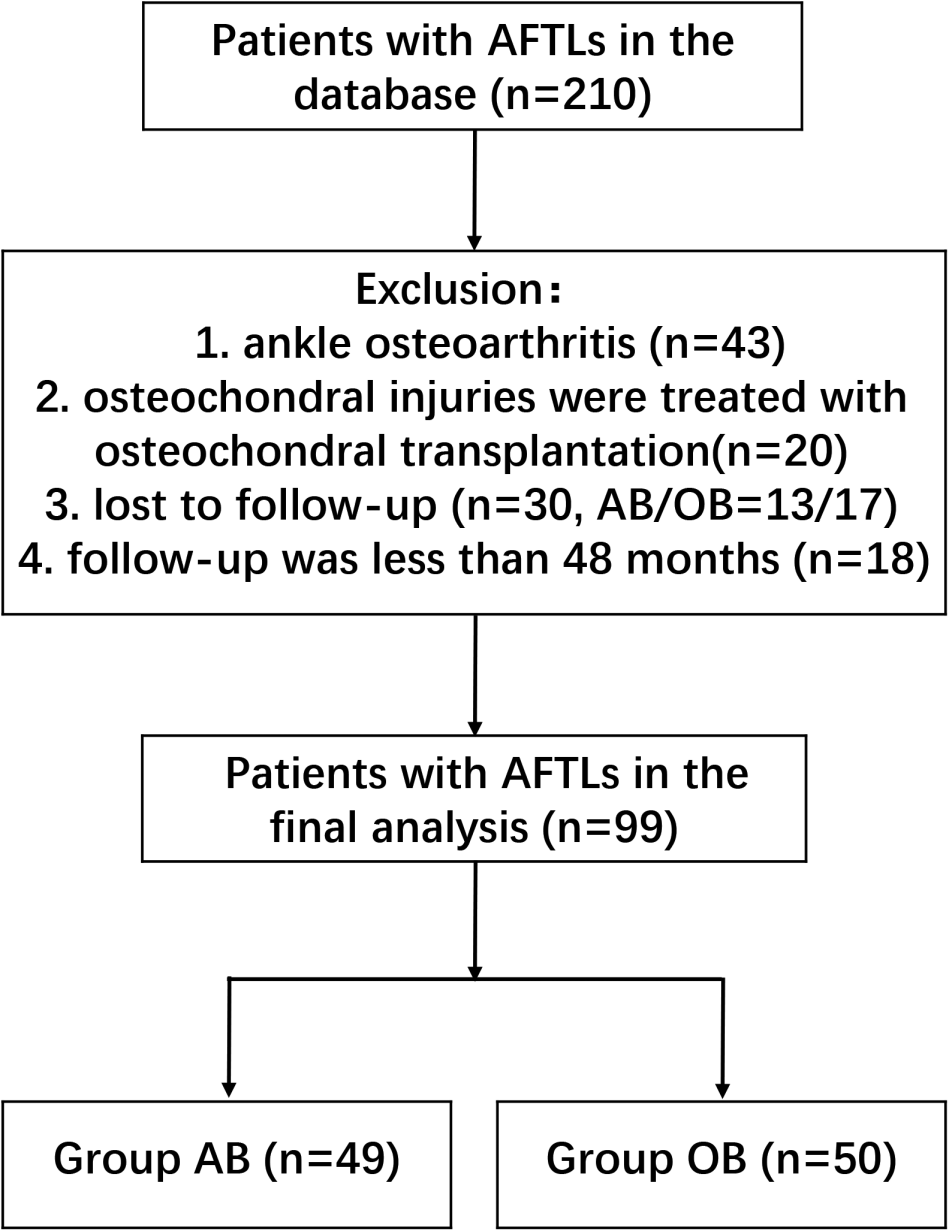
Flow chart of patients in this study.

The inclusion criteria were: (1) MRI confirmed the tear, unconsolidated of the AFTL; (2) recurrent ankle sprain and signs of ankle instability: the talar tilt test was greater than 5° under x-ray, the anterior drawer test was positive; no previous ankle ligament surgery, with no other ankle injury than the index one; (3) complete surgical and follow-up data, and follow-up for at least 48 months. The exclusion criteria were: (1) Severe ankle osteoarthritis or osteochondral injury requiring osteochondral transplantation; (2) severe underlying disease and inability to tolerate surgery. A total of 210 patients with AFTLs admitted to our hospital from June 2014 to June 2018 were enrolled. A single independent investigator (VP) who was not engaged in the treatment of the patients used computer-generated randomization to assign individuals to either the arthroscopic Broström-Gould technique or the open Broström-Gould technique. The following patients were not eligible for the study: 43 patients had severe ankle osteoarthritis, 20 patients with osteochondral injuries were treated with osteochondral transplantation, 30 patients were lost to follow-up, including 13 patients in the AB group and 17 patients in the OB group, and 18 patients were followed up for less than 48 months. Eventually, a total of 99 patients with AFTLs were included. Of whom 49 patients underwent arthroscopic Broström-Gould technique, while the other 50 patients underwent open Broström-Gould technique (Figure 1). This study was approved by our hospital Ethics Committee.

### 2.2. Perioperative management

Before surgery, all patients were given health education about disease and operation to eliminate perioperative care nervousness, speed up patients with recovery and even improve our work efficiency. And prophylactic antibiotic (cefuroxime sodium 1.5 g) were started 30 min before surgery.

During operation, the two groups underwent surgery under spinal anesthesia or general anesthesia. For both surgical treatments, all operations were performed by a same group of surgeons, which were expert technicians and well experienced. All the patients underwent an arthroscopic evaluation of the ankle joint under standard anteromedial (AM) and anterolateral portal (AL). The intra-articular lesions (including osteochondral defect of the talus and anterior impingement syndrome by osteophyte) were fully evaluated and managed.

Arthroscopic Broström-Gould operation was conducted by modifying Nery's study (14). Arthroscopic exploration was performed to examine the ATFL, expose and debride the ligament attachment point on the fibula. Then, a double-loaded suture anchor [4.5 mm polyether ether ketone (PEEK) anchor, Arthrex] was inserted into the mid-portion of the attachment area of the fibula. The ATFL and lateral retinaculum were sutured with tail suture. The ankle joint was in a neutral position, and the suture was tied. The test of the anterior drawer was negative after suturing. After suturing the wound, a short leg cast was applied in the neutral position.

The open Broström-Gould procedure was performed by the standard surgical method, which was then also repaired with a double-loaded suture anchor (4.5 mm PEEK anchor, Arthrex). The test of the anterior drawer was negative after suturing. After suturing the wound, a short leg cast was applied in the neutral position.

After surgery, the patients of two groups received antibiotics (cefuroxime 750 mg three times daily) to prevent infection not more than 48 h. Two weeks after the surgery, the short leg cast was changed to an ankle brace, then strengthening exercises of the whole lower extremity and flexion and extension of joint were encouraged. After 6 weeks, physical activities (balance training and full weightbearing) were encouraged.

### 2.3. Postoperative follow-up and observational indexes

The surgery duration, hospitalization time, and postoperative complications were assessed after surgery, and ADT, VAS scores, AOFAS scores, K-P scores, and Tegner activity scores were evaluated to assess ankle function. AOFAS and K-P scores are commonly used in clinical evaluation of ankle joint function. Tegner activity score was first characterized as an activity level assessment system for knee joints; however, it has also been used to assess activity following ankle damage or surgery (8, 15–17). All measurements were made at 6 months, 12 months, 24 months and 48 months after surgery, and clinically evaluated by the same experienced rehabilitation physician who did not take part in surgery and was unaware of how the surgery was carried out.

### 2.4. Statistical analyses

For statistical studies, the SPSS 23.0 program (IBM Corp, Armonk, NY, USA) was utilized. All data were evaluated for homogeneity of normality and variance using Shapiro-Wilk test and Levene's test, respectively. The independent sample *t* test was used for comparing continuous variables with homogeneity of variance and normality between groups. Otherwise, Mann-Whitney U test or Wilcoxon signed-rank test for nonparametric data. Pearson's *χ*^2^ test were used to compare categorial variables in our study. The significance level was set at *P* < 0.05.

## 3. Results

### 3.1. Comparison of baseline characteristics

A total of 99 patients with chronic lateral ankle instability were included in this study, including 66 males and 33 females. The mean age of the patients in the group AB and the group OB was 31.71 ± 4.99 years and 31.92 ± 4.77 years, respectively, the age distribution of the two groups has no statistical sense (*P* = 0.83). No significant differences were found regarding sex (*P* = 0.57), body mass index (BMI) (*P* = 0.25), or side (left/right) (*P* = 0.75) between group AB and group OB. 39 of the patients in the arthroscopic group showed synovitis, seven had osteochondral defects (OCD), and three had osteophytes. 42 patients in the open group had showed synovitis, ten had OCD, while five others had osteophytes. There were no discernible difference in the incidence of synovitis (79.59% vs. 84%; *P* = 0.57), OCD (14.3% vs. 20%; *P* = 0.45) or osteophyte (6.1% vs. 10%; *P* = 0.48) between group AB and group OB. Ankles with OCD underwent chondroplasty by microfracture, while debridement was carried out on those patients who had osteophytes. The symptom duration was 25.0 ± 8.48 months (range, 24–40) in the group AB and 23.52 ± 8.37 months (range, 26–45) in the group OB. There was also no statistically significant difference in symptom duration between the 2 groups (*P* = 0. 38). The method of anesthesia and mean follow-up period did not differ between the 2 groups (*P* = 0. 51, *P* = 0. 14). Operation time and mean hospitalization time were similar between the two groups, with no statistically significant difference (43.58 ± 16.61 min vs. 48.45 ± 12.76 min, 6.41 ± 2.57 days vs. 6.70 ± 2.72 days, *P* > 0.05) (Table 1).

**Table 1:** Demographic characteristics of patients.

Variable	AB	OB	Statistical analyses	*P*
Age (years)	31.71 ± 4.99	31.92 ± 4.77	*T* = 0.210	0.83
Gender			*χ*^2^ = 0.323	0.57
Female	17	16		
Male	32	34		
BMI (kg/m^2^)	21.84 ± 2.40	22.38 ± 2.29	*T* = 1.151	0.25
Side (left/right)	21/28	23/27	*χ*^2^ = 0.099	0.75
Symptom duration (months)	25.0 ± 8.48	23.52 ± 8.37	Wilcoxon *W* = 2,367.5	0.35
**Comorbidities**				
Synovitis	79.6%	84%	*χ*^2^ = 0.323	0.57
osteochondral defects (OCD)	14.3%	20%		0.45
osteophyte	6.1%	10%		0.48
Anesthesia (spinal/general)	42/7	45/5	*χ*^2^ = 0.427	0.51
Operation time (min)	43.58 ± 16.61	48.45 ± 12.76	*T* = −1.633	0.11
Hospitalization time (days)	7.41 ± 2.57	7.70 ± 2.72	Wilcoxon *W* = 2,372.5	0.58
Mean follow-up (months)	52.69 ± 3.66	54.08 ± 4.62	Wilcoxon *W* = 2,240	0.14

Data presented as the mean ± SD or *n* (%), and *P* < 0.05 was considered to indicate a statistically significant difference.

### 3.2. Comparison of clinical outcomes

**Figure 2:**
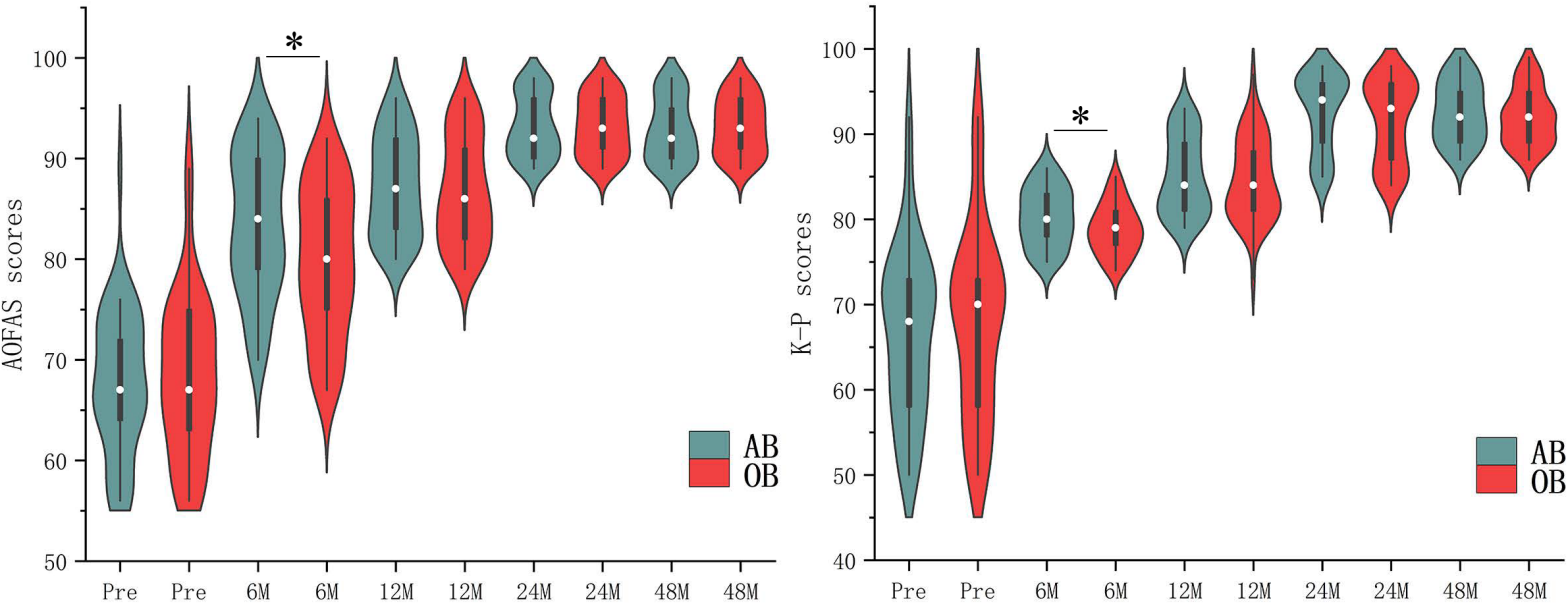
Comparisons of key patient data in Table 2.

As shown in Table 2 and Figure 2, the preoperative ADT, VAS scores, AOFAS scores, K-P scores, and Tegner activity scores were comparable in both the open and arthroscopic groups (all *P* > 0.05). Postoperatively, ADT was comparable in both and improved significantly from preoperative values. After surgery, a significant increase was noted in AOFAS scores (from 67.59 ± 7.02 to 92.90 ± 3.14), K-P scores (from 57.75 ± 9.93 to 92.55 ± 3.52), and Tegner activity scores (from 1.9 ± 0.58 to 6.31 ± 1.28), and a significant decrease in VAS scores (from 6.12 ± 1.90 to 2.12 ± 1.05) in group AB as well as the group OB (AOFAS scores from 68.34 ± 7.99 to 93.20 ± 2.86; K-P scores from 58.82 ± 11.07 to 92.02 ± 3.29; Tegner activity scores from 1.68 ± 0.70 to 5.82 ± 1.42; VAS score from 6.48 ± 1.78 to 2.52 ± 1.17). However, no significant difference were found in ADT, VAS scores, AOFAS scores, K-P scores, and Tegner activity scores between the two groups at the final follow-up (*P* > 0.05 for all variables). Especially in the early term follow-up (6 months), group AB had significantly higher AOFOS and K-P scores compared with the group OB at the 6 months postoperatively (*P* < 0.05).

**Table 2:** Clinical examinations of patients.

Variable	AB	OB	Statistical analyses	*P*
**ADT**				
Preoperative	6.90 ± 1.48	6.72 ± 1.80	Wilcoxon *W* = 2,411.5	0.53
48 months postoperatively	5.33 ± 1.40*	5.54 ± 1.54*	Wilcoxon *W* = 2,355.5	0.50
**VAS scores**				
Preoperative	6.12 ± 1.90	6.48 ± 1.78	*T* = 0.986	0.34
48 months postoperatively	2.12 ± 1.05*	2.52 ± 1.17*	*T* = 1.869	0.66
**Tegner activity scores**				
Preoperative	1.90 ± 0.58	1.68 ± 0.70	*T* = −1.849	0.68
48 months postoperatively	6.31 ± 1.28*	5.82 ± 1.42*	*T* = −1.786	0.77
**AOFAS scores**				
Preoperative	67.59 ± 7.02	68.34 ± 7.99	*T* = 0.495	0.62
6 months postoperatively	83.45 ± 7.07*	79.74 ± 7.35*	*T* = −2.558	**0**.**01**
12 months postoperatively	87.24 ± 4.96*	86.70 ± 5.30*	*T* = −0.527	0.59
24 months postoperatively	93.18 ± 3.10*	93.22 ± 2.92*	Wilcoxon *W* = 2,439.5	0.94
48 months postoperatively	92.90 ± 3.14*	93.20 ± 2.86*	*T* = 0.501	0.62
**K-P scores**				
Preoperative	57.75 ± 9.93	58.82 ± 11.07	*T* = 0.503	0.62
6 months postoperatively	80.27 ± 3.46*	78.80 ± 2.97*	*T* = −2.264	**0**.**03**
12 months postoperatively	85.04 ± 4.52*	84.62 ± 4.84*	Wilcoxon *W* = 2,436	0.65
24 months postoperatively	92.61 ± 4.49*	91.48 ± 4.77*	Wilcoxon *W* = 2,312	0.19
48 months postoperatively	92.55 ± 3.52*	92.02 ± 3.29*	*T* = −0.776	0.44

Data presented as the mean ± SD or *n* (%), and Bold highlights of the *P* value (*P* < 0.05) was considered to indicate a statistically significant difference.

* *P* < 0.05 vs. respective preoperative data.

### 3.3. Comparison of complications

The incidence of postoperative complications in group AB was higher than that in group OB, although not statistically significant (16.3% vs. 14%, *P* = 0.75) (Table 2). In the group AB, the surgical complications occurred in eight patients, which include six patients reported sensory disturbance on the lateral aspect of the foot (superficial peroneal nerve injuries), and two patients with superficial wound infection. In the group OB, the surgical complications were observed in seven patients, which include two patients with superficial peroneal nerve injuries, and five patients with superficial wound infection. Superficial wound infection is defined as infection of the wound, in which there is no evidence that the infection extends to the site of the implant. The patients with superficial infection were treated successfully by antibiotics and recovered without any influence on the final result. The patients with superficial peroneal nerve injuries were completely asymptomatic, pain free, and stable at the last follow-up. None of the patients in either group required secondary operations within 48 months.

## 4. Discussion

The main finding of the present study is that the arthroscopic Broström-Gould technique is comparable to open Broström-Gould procedure in ATFL repair for chronic lateral ankle instability. The postoperative evaluation with a minimum of 48 months of follow-up indicated that the two surgical procedures can produce similar functional and clinical outcomes, and both of them were effective treatments for chronic lateral ankle instability. As we all know, the open Broström-Gould surgery is frequently used to treat chronic lateral ankle instability, and provide excellent mid-term results. Maffulli et al. found that open Broström-Gould surgery enhanced the AOFAS scores from 51 to 90 points following a 9-year follow-up (18). Buerer et al. demonstrated that the AOFAS scores was 89 at the last follow-up in 41 patients following open Broström-Gould repair, with a high satisfaction rate (19). Molloy et al. found that in 21 patients with persistent lateral ankle instability who had open Broström-Gould repair, the preoperative AOFAS scores of 53 improved to 89 at 25 months following surgery (20). Our results showed that in the 50 patients receiving the open Broström-Gould repair, the AOFAS score was improved from 65.4 ± 9.1 (preoperative) to 91.1 ± 6.2 (at 4 years after surgery), while the K-P score was improved from 60.5 ± 9.8 to 90.5 ± 8.8, suggesting that the open Broström-Gould repair has good therapeutic efficacy, which is consistent with previous studies.

Recently, many authors have proposed performing the Broström-Gould procedure completely with arthroscopy (9, 21, 22). With a mean follow-up of 9.8 years, Nery et al. documented the long-term outcomes of arthroscopic ankle stabilization in 38 patients, whose got the mean AOFAS score was 90 at the last follow-up (14). Cottom and Rigby also showed similar results in 40 patients with a mean follow-up of 12.3 months and a mean AOFAS score of 95.4 at the last follow-up compared with a mean preoperative score of 41.2 (23). In this study, for the 49 cases of arthroscopic repair of the ATFL, the AOFAS score improved from 63.1 ± 9.1 (preoperative) to 92.4 ± 6.0 (at 48 months after surgery) and the K-P score improved from 59.6 ± 9.7 to 89.2 ± 8.4. All of these data indicate that arthroscopic ATFL repair has a high therapeutic effectiveness. These and other trials suggest that arthroscopic stabilization is a successful treatment with good to outstanding outcomes. However, studies comparing arthroscopic and open Broström-Gould procedures are still limited, and most of these studies are limited to small sample sizes and short-term follow-up. To the best of our knowledge, this study is the first to compare the two surgical procedures with lager sample sizes and a relatively long follow-up period than previous studies. A total of 99 cases were included in the present study, which compared the mid-term (48M) therapeutic efficacy between the arthroscopic repair and open Broström-Gould repair of the ATFL for chronic lateral ankle instability. The postoperative ADT, VAS, AOFAS scores, K-P scores, or Tegner activity scores were significantly improved compared to the preoperative baselines, and there was no significant difference in these parameters between the two groups at final follow-up. This indicated that arthroscopic repair could be a viable alternative to the open Broström-Gould repair procedure for chronic lateral ankle instability.

Compared with open surgery, arthroscopic surgery has the own advantages of small incision and less invasiveness, but whether it can lead to better early clinical functional outcomes. Reviewed published research findings, the early postoperative period outcomes are controversial. In a randomized controlled experiment, Yeo et al. discovered no statistically significant changes in the VAS, AOFAS, and Karlsson scores between the open and arthroscopic modified Broström procedures at short-term follow-up (6 weeks and 6 months) (9). On the other hand, Kentaro Matsui et al. reported that the arthroscopic procedure resulted in less postoperative pain and an earlier return to daily activities (24). Lower perioperative VAS and higher AOFAS scores at the 6 and 12 months follow-ups were two benefits of the arthroscopic Brostrom-Gould approach, according to Bo Jun Woo et al. (11). Recently, at 6 months after surgery, Zongchen Hou et al. discovered that the arthroscopic group had seen a shorter recovery duration, greater rates of return to athletic activities, and improved clinical results, posture control, and muscular strength (25). Attia, A. K et al. performed meta-analysis using one RCT, one PCS and six RCTs including a total of 408 patients with chronic lateral ankle instability to prove that arthroscopic Broström was superior to open Broström-Gould surgery in postoperative AOFAS scores, VAS pain scores, and time to return to weightbearing (26). Consistent with those of the above three studies, the present results showed that the arthroscopic group had better outcomes (AOFOS and K-P scores) than the open group at 6 months follow-up. We speculated that the phenomenon might be related to the following reasons. First, the higher functional scores in the arthroscopic group can be attributed to the smaller amount of soft tissue dissection required for arthroscopic operations. The open group, however, required a longer period of recovery because of the more severe trauma caused by the surgery. Second, compared with the open procedure, the arthroscopic procedure prevents injury of blood vessels around the ATFL and facilitates vascularization of the repaired ATFL.

According to existing research results (2, 26, 27), there is a trend for a higher rate of superficial peroneal nerve complications in arthroscopic surgery and a higher rate of wound complications in open surgery, which is consistent with the findings of our study. A systematic review by Guelfi et al. reported excellent clinical outcomes and patient satisfaction rates, but a high complication rate of 15.27% associated with arthroscopic Broström procedures (5). The incidence rate for our arthroscopic procedure was 12.2% (6/49), which was within an acceptable range. The possibility of nerve injury was linked to portal proximity, nerve anatomical variations, and nerve entrapment during the procedure. It is critical to identify and mark anatomic landmarks prior to surgery to reduce the risk of nerve injury. Prior to making incisions in the lateral ankle region, the arthroscopic “safe zone,” which includes the distal fibula tip, the superior margin of the peroneal tendons, and the intermediate branch of the superficial peroneal nerve, should be marked (28). Then, using a small hemostat, make an incision only through the thickness of the skin, followed by dissecting subcutaneous tissues. When inserting tools into the joint, it is also recommended to use a cannula to minimize unintended nerve entrapment. As experience progresses and surgical technique evolves, we believe that continuous improvement in efficiency and complication rates may be predicted.

The limitations of the study include the following: (1) This study was a retrospective study and the significance of the data between scores and actual function may vary with experience. However, a double-blind randomized controlled trial may not be suitable for many patients in China because of the medical risk. When it comes to participating in double-blind randomized controlled trials (RCTs), most patients may not trust their doctors. And most patients with chronic ankle instability have an understanding of surgical treatments through multiple outpatient clinical visits and online inquiries before surgery. These may be not conducive to a double-blind RCT. However, we always believe that RCTs are necessary, and we'll overcome the related difficulties to conduct RCTs in the future. Second, only the ATFL was repaired in this investigation. For patients with calcaneofibular ligament injury, it still unknown if the untreated calcaneofibular ligament affects clinical efficacy in the long-term follow up needs to be further studied. Third, postoperative assessment is mostly based on functional measures. Objective quantitative data are sparse, particularly MRI data included radiographic signs of degenerative changes preoperatively and postoperatively from recruited patients.

## 5. Conclusion

In conclusion, our results suggest that arthroscopic and open Broström-Gould repair of the ATFL have comparable therapeutic efficacy for chronic lateral ankle instability in the mid-term follow-ups. No significant differences were found statistically regarding complications between two groups. The arthroscopic Broström-Gould procedure had higher AOFAS and K-P scores at 6 months after operation.

## Data Availability

The original contributions presented in the study are included in the article, further inquiries can be directed to the corresponding author.
